# Comparative Analysis of Volatile Compounds and Characterization of Key Flavor Compounds in *Cinnamomum cassia* Barks of Different Cultivars

**DOI:** 10.3390/foods15040723

**Published:** 2026-02-15

**Authors:** Jing Chen, Libing Long, Ying Zhu, Liujun Chen, Linshuang Li, Ding Huang, Ruhong Ming, Rongshao Huang, Jian Xiao, Shaochang Yao

**Affiliations:** 1 College of Pharmacy, Guangxi University of Chinese Medicine, Nanning 530200, China; chenjing2023@stu.gxtcmu.edu.cn (J.C.); longlibing2023@stu.gxtcmu.edu.cn (L.L.);; 2 Guangxi Engineering Research Center for High-Value Utilization of Guangxi-Produced Authentic Medicinal Herbs, Guangxi University of Chinese Medicine, Nanning 530200, China; 3 University Engineering Research Center of Characteristic Traditional Chinese Medicine and Ethnomedicine, Guangxi University of Chinese Medicine, Nanning 530200, China

**Keywords:** *Cinnamomum cassia*, HS-SPME-GC-MS, flavor compounds, rOAV, phenotypic traits, PCA

## Abstract

Consumer demand is growing for traceable, high-quality *Cinnamomum cassia* with defined sensory attributes. However, research linking cultivar morphology to these specific flavor signatures remains scarce. This study elucidated the relationships between phenotypic traits, volatile constituents, and key aroma characteristics of three *C. cassia* cultivars (Xijiang [XJ], Dongxing [DX], and Qinghua [QH]) using phenotypic evaluation, headspace solid-phase microextraction–gas chromatography–mass spectrometry (HS-SPME-GC-MS), and a combination of relative odor activity value and principal component analysis (rOAV-PCA). XJ exhibited an intensely spicy aroma, attributable to its high *trans*-cinnamaldehyde content (718.76 ± 60.08 mg/g). In contrast, DX showed the highest δ-cadinene level (44.86 ± 4.48 mg/g) and a complex spicy–woody–sweet profile, shaped by sesquiterpenes such as α-humulene, α-copaene, caryophyllene, and β-caryophyllene. QH displayed both a high volatile oil yield (2.57 ± 0.28%) and a distinct herbal–woody character, primarily contributed by δ-cadinene and α-muurolene. This study constructed an integrated phenotype–chemistry–sensory map for *C. cassia* cultivars, facilitating cultivar discrimination, supporting flavor quality management, and enabling marker-assisted breeding for desirable aroma profiles.

## 1. Introduction

Cortex Cinnamomi (“Rougui”), the dried bark of *Cinnamomum cassia* Pres, is a high-value spice and medicinal herb with substantial commercial potential [1]. Over 160 chemical compounds (volatile: cinnamaldehyde and sesquiterpenes; non-volatile: flavonoids and alkaloids) have been isolated [2], exhibiting antioxidant, anti-inflammatory, antibacterial, and anti-tumor activities [3,4,5,6,7]. However, its food industry value is primarily determined by unique flavor profiles, a key factor driving market competitiveness amid surging global demand for premium natural spices. Thus, scientifically characterizing and distinguishing “Rougui” aroma across dominant cultivars is critical, as current knowledge in this field remains incomplete and fails to fully support industrial applications, requiring integrated instrumental and sensory evaluation techniques.

*C. cassia* is mainly cultivated in Guangxi, Guangdong, and Fujian (China), accounting for over 90% of national output [8,9]. Three dominant commercial cultivars are widely grown: “Xijiang” (XJ), “Dongxing” (DX), and “Qinghua” (QH) [1,10,11], with QH renowned for its intense aroma and superior commercial potential [11]. Despite their economic significance, there is a critical lack of systematic investigations into the divergence of their volatile compound profiles and key flavor compounds, a gap that hinders targeted breeding and standardized commercial cultivation for quality improvement.

The Chinese Pharmacopeia (ChP) designates volatile oils (>1.2%) and cinnamaldehyde (>1.5% DW) as “Rougui” quality markers [11,12], with cinnamaldehyde and terpenoids being the major volatile components [2,7]. Five quality markers, including cinnamaldehyde, coumarin, cinnamyl alcohol, cinnamic acid, and *o*-methoxy cinnamic aldehyde, were proposed as quality markers (Q-Markers) to analyze the quality differences of *C. cassia* [13]. A total of 72 phenylpropanoids, 146 flavonoids, and 130 terpenoids were detected by metabolomic analyses of 5~8 years old *C. cassia* [14]. Our previous study identified 28 aroma-related VOCs with harvest-time accumulation [15]. Given aroma is a core quality determinant impacting preference [16], elucidating cultivar-specific VOC profiles is urgently needed to provide actionable guidance for targeted cultivation and breeding practices.

Notably, cinnamon aroma is not solely dictated by cinnamaldehyde [9]; trace terpenoids, esters, and aldehydes shape flavor due to their low odor thresholds [17,18]. Headspace solid-phase micro extraction–gas chromatography–mass spectrometry (HS-SPME-GC-MS) combined with the relative odor activity value (rOAV) and principal component analysis (PCA) is a robust tool for identifying key flavor compounds and quantifying inter-cultivar differences [17,19,20,21,22,23,24,25], providing a reliable analytical framework for testing hypotheses about cultivar-specific flavor traits.

To address the aforementioned knowledge gaps and move beyond descriptive analyses, we hypothesized that XJ, DX, and QH exhibit distinct morphological, physiological, and volatile metabolic profiles, and that their cultivar-specific key flavor compounds can be systematically identified via HS-SPME-GC-MS coupled with rOAV and PCA. To test this hypothesis, this study aims to characterize and differentiate the three cultivars’ traits, identify their unique key flavor compounds, and provide evidence-based insights for improving *C. cassia* aromatic quality via targeted cultivation and breeding strategies, thereby bridging the gap between scientific research and industrial application.

## 2. Materials and Methods

### 2.1. Plant Materials and Reagents

A total of 78 bark samples were collected in April 2025 from seven-year-old *C. cassia* plants originated from Guangxi, Guangdong, Fujian, and Yunnan provinces in China, which belonged to three commercially predominant cultivars with XJ (*n* = 45), DX (*n* = 9), and QH (*n* = 24) (Table S1). These materials were identified by Professor Rongshao Huang from Guangxi University of Chinese Medicine. The voucher specimens were deposited in the University herbarium (No. 005382). The leaves and bark (10 cm above ground) were obtained from three individuals and pooled into a single sample to minimize biological variability. All the samples were frozen in liquid nitrogen immediately after collection and stored at −80 °C for subsequent analysis. All analytical grade reagents were purchased from Sinopharm Medicine Holding Co., Ltd. (Shanghai, China). All standards and chromatographic-grade reagents were purchased from Sigma-Aldrich (St. Louis, MO, USA).

### 2.2. Determination of Morphological and Physiological Traits

The bark and leaf thickness were measured using a digital electronic caliper with a precision of 0.01 mm. Leaf width and length were measured by ruler, and their ratio was calculated. The dry rate (%) was calculated by the following Formula (1), where *W_dry_* = dry weight after oven-drying at 50 °C to constant weight; *W_fresh_* = fresh weight of the sample. The soluble sugar, soluble protein, and flavonoid content were determined according to Gao [11]. The content of total volatile oil, *trans*-cinnamaldehyde, and δ-cadinene were determined following our previous methods [15,26].

(1) $$\mathrm{Dry}\;\mathrm{rate}(\%)=\frac{W_{\mathrm{dry}}}{W_{fresh}}\times 100$$

### 2.3. HS-SPME-GC-MS Analysis

Volatile compounds were analyzed by HS-SPME-GC-MS analysis, with semi-quantitative results expressed as internal standard equivalents following the protocols as described in our previous study [15]. Briefly, 0.5 g of finely ground dried bark powder was accurately weighed into a 20 mL headspace vial (Agilent, Palo Alto, CA, USA) with 2 mL of saturated NaCl solution and 10 μL of n-butane (0.05 mg/mL in methanol) as the internal standard before sealing with a TFE-silicone septum (Agilent). The vial was equilibrated at 60 °C for 5 min; then, a 120 μm divinylbenzene/carboxen/polydimethylsiloxane (DVB/CAR/PDMS) fiber was used for volatile adsorption at 60 °C for 15 min, followed by thermal desorption at 250 °C for 5 min in splitless mode.

The identification and quantification of VOCs were carried out using an Agilent Model 8890 GC and a 7000D mass spectrometer (Agilent, Palo Alto, CA, USA), equipped with a DB-5MS capillary column (30 m × 0.25 mm × 0.25 μm). High-purity helium (99.999%) was used as carrier gas at a flow rate of 1.2 mL/min. The injector temperature was kept at 250 °C and the detector at 280 °C. The oven temperature program: 40 °C (3.5 min) → 100 °C (10 °C/min) → 180 °C (7 °C/min) → 280 °C (25 °C/min, for 5 min). Mass spectra were recorded in electron impact (EI) ionization mode at 70 eV. The quadrupole mass detector, ion source, and transfer line temperatures were set, respectively, at 150, 230, and 280 °C. The MS was in selected ion monitoring (SIM) mode and was used for the identification and quantification of analyses. MassHunter quantitative analyses and internal standard normalization were used to calculate the peak area and relative content of each compound. Compounds were identified by matching with the NIST 2023 library (match factor ≥ 800) and retention index comparison.

### 2.4. Calculation of Relative Odor Activity and Analysis of Odor Characteristics

The rOAV value was utilized to identify key flavor compounds by integrating their concentrations with sensory thresholds, thereby highlighting the specific contribution of individual aroma components to the overall flavor profile. Generally, compounds exhibiting an rOAV ≥ 1 are considered to have a significant influence on flavor perception. Following the protocol described by Huang [27], where *C_i_* represents the relative content of compound i (%) and T_i_ denotes its sensory threshold (mg/L), the rOAV was calculated according to the following Formula (2). The threshold values and the odor type were obtained from the previous literature [22,28,29,30,31,32,33,34,35,36,37,38,39]. According to the reference [21], the odor characteristics of each compound were classified as follows: Class A: fresh and green scents; Class B: floral, fruity, and sweet scents; Class C: herbal and wood scents; Class D: a bake scent; and Class E: an unpleasant scent (Table S2).

(2) $$rOAV_{i}=\frac{C_{i}}{T_{i}}$$

### 2.5. Statistical Analysis

All experiments were performed with three biological replicates and three technical replicates, and results are expressed as mean ± standard deviation (SD). One-way analysis of variance (ANOVA), followed by Duncan’s multiple range test (*p* < 0.05), was performed using IBM SPSS Statistics 27.0 software. The standardized metabolite data were imported into the Metware Cloud platform https://cloud.metware.cn (accessed on 12 November 2025) for visualization and analysis. PCA and hierarchical cluster analysis (HCA) were performed, and clustering results were visualized as heatmaps to illustrate metabolite accumulation patterns across different cultivars. Differential metabolites between groups were identified based on variable importance in projection (VIP) values (VIP > 1) obtained from orthogonal partial least squares discriminant analysis (OPLS-DA) and absolute Log_2_ fold change (|Log_2_FC| ≥ 1.0). Values marked with different lowercase letters are significantly different (*p* < 0.05).

## 3. Results

### 3.1. Distribution of C. cassia Cultivars

**Figure 1:**
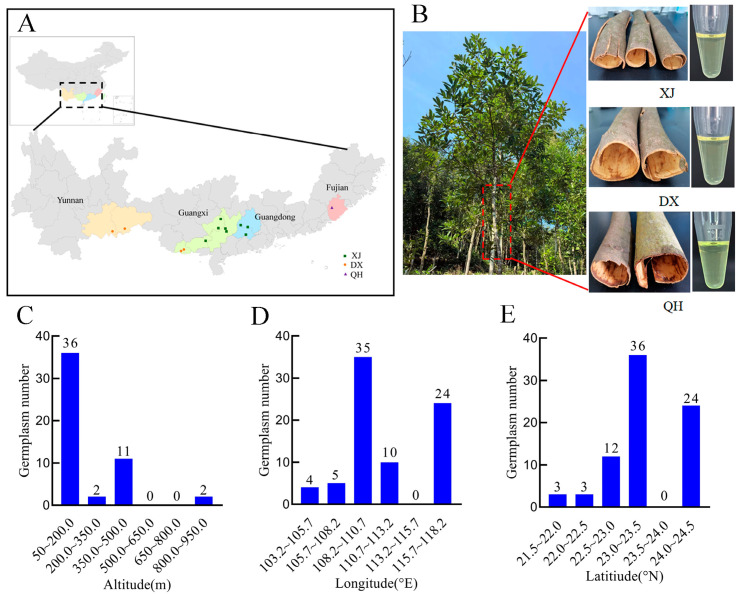
Sampling characteristics of *C. cassia* germplasm. (**A**) Sampling site distribution map, with solid dots representing the geographic locations of *C. cassia* germplasm collection sites; (**B**) phenotypes of bark and volatile oil of the three *C. cassia* cultivars; (**C**) altitude distribution; (**D**) longitude distribution; and (**E**) latitude distribution. Abbreviations: XJ, Xijiang; DX, Dongxing; QH, Qinghua.

The material consisted of three commercially predominant cultivars of *C. cassia*: XJ (*n* = 45; 57.69%), DX (*n* = 9; 11.54%), and QH (*n* = 24; 30.77%) (Figure 1A). Visual assessment revealed no distinct morphological differences in bark among the three cultivars, whereas the color of volatile oils varied slightly, with the QH sample exhibiting a brighter yellow appearance (Figure 1B). All sampling sites were distributed at an altitude of 57.30 to 862.10 m above sea level (m a.s.l.), with the majority of accessions (63 samples, 80.77%) located in low-hill areas below 200 m a.s.l. (Figure 1C). The samples collection covered a geographic range of 103.84–117.30° E longitude and 21.66–24.54° N latitude, with the highest sampling density observed at 108.2–110.7° E (*n* = 35) and 22.5–23.5° N (*n* = 36) (Figure 1D,E). Collectively, these sampling characteristics confirm that the germplasm panel in the present study encompasses the primary commercial cultivation regions of *C. cassia* in China, thus ensuring the broad representativeness of the experimental samples.

### 3.2. Phenotypic Divergence in C. cassia Cultivars

**Figure 2:**
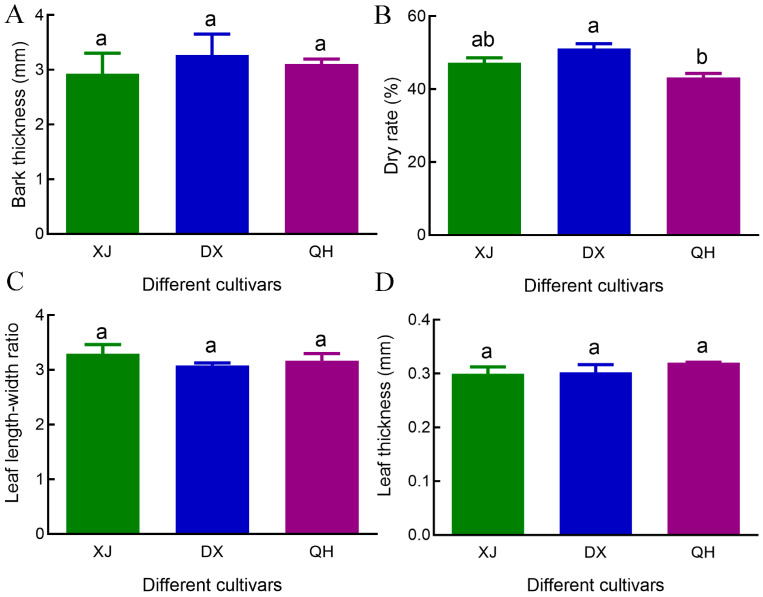
Phenotypic variation in *C. cassia* cultivars. (**A**) Bark thickness. (**B**) Drying rate. (**C**) Leaf length-to-width ratio. (**D**) Leaf thickness. Different lowercase letters above bars indicate significant differences at *p* < 0.05, and bars sharing the same letter indicate no statistically significant differences. Abbreviations: XJ, Xijiang; DX, Dongxing; QH, Qinghua.

Phenotypic evaluation revealed variations among the three cultivars. While bark thickness was numerically the greatest in DX (3.27 ± 0.39 mm), followed by QH (3.11 ± 0.09 mm) and XJ (2.93 ± 0.38 mm), these differences were not statistically significant (Figure 2A). DX exhibited the highest drying rate (51.12 ± 1.34%), which was significantly higher than that of QH (43.20 ± 1.10%; *p* < 0.05); XJ (47.22 ± 1.40%) showed an intermediate drying rate with no significant difference from either cultivar (Figure 2B). For leaf morphological traits, neither the leaf length-to-width ratio nor leaf thickness differed significantly among the three cultivars (Figure 2C,D).

### 3.3. Physiological and Biochemical Profiles of C. cassia Cultivars

**Figure 3:**
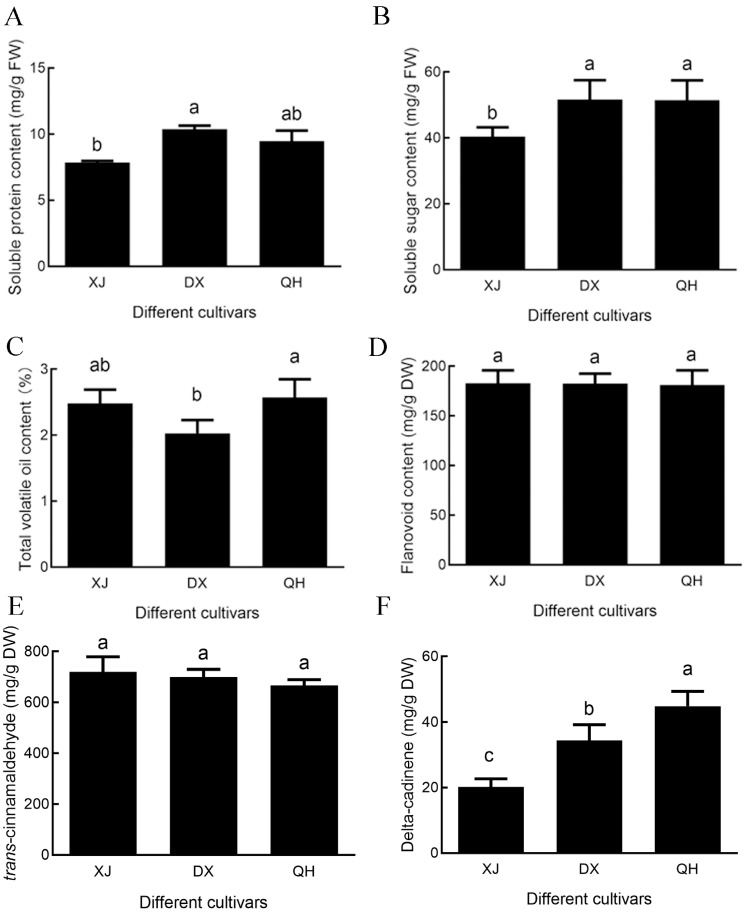
Physiological and biochemical profiles of *C. cassia* cultivars. (**A**) Soluble protein content. (**B**) Soluble sugar content. (**C**) Total volatile oil content. (**D**) Total flavonoid content. (**E**) *trans*-Cinnamaldehyde content. (**F**) δ-Cadinene content. Different lowercase letters above bars indicate significant differences at *p* < 0.05, and bars sharing the same letter indicate no statistically significant differences. Abbreviations: XJ, Xijiang; DX, Dongxing; QH, Qinghua.

The soluble protein content of DX (10.37 ± 0.27 mg/g) was significantly higher than that of XJ (7.85 ± 0.14 mg/g) (*p* < 0.05) (Figure 3A). Similarly, the soluble sugar content of DX (51.63 ± 5.88 mg/g) was significantly higher than that of XJ (40.41 ± 2.81 mg/g) (*p* < 0.05) (Figure 3B). However, it is worth noting that the total volatile oil content of DX was the lowest among the three cultivars, which was significantly lower than that of QH (*p* < 0.05) (Figure 3C). In contrast, no significant differences in total flavonoid content were detected across the three cultivars (XJ: 182.88 mg/g; DX: 182.67 mg/g; and QH: 181.33 mg/g) (Figure 3D).

*Trans*-cinnamaldehyde and δ-cadinene were quantified via an external standard method using GC-MS (Figure S1). The calibration curves showed excellent linearity for *trans*-cinnamaldehyde (0.0813–16.2680 mg/mL, *R*^2^ = 0.99977) and δ-cadinene (0.0068–1.3500 mg/mL, *R*^2^ = 0.99906) (Figure S2). No significant differences in *trans*-cinnamaldehyde content were observed among the cultivars (Figure 3E), whereas δ-cadinene content differed significantly (*p* < 0.05), with the highest level in QH (44.86 ± 4.48 mg/g) and the lowest in XJ (20.21 ± 2.47 mg/g) (Figure 3F).

### 3.4. Differential Accumulation of Volatile Compounds in C. cassia Cultivars

**Figure 4:**
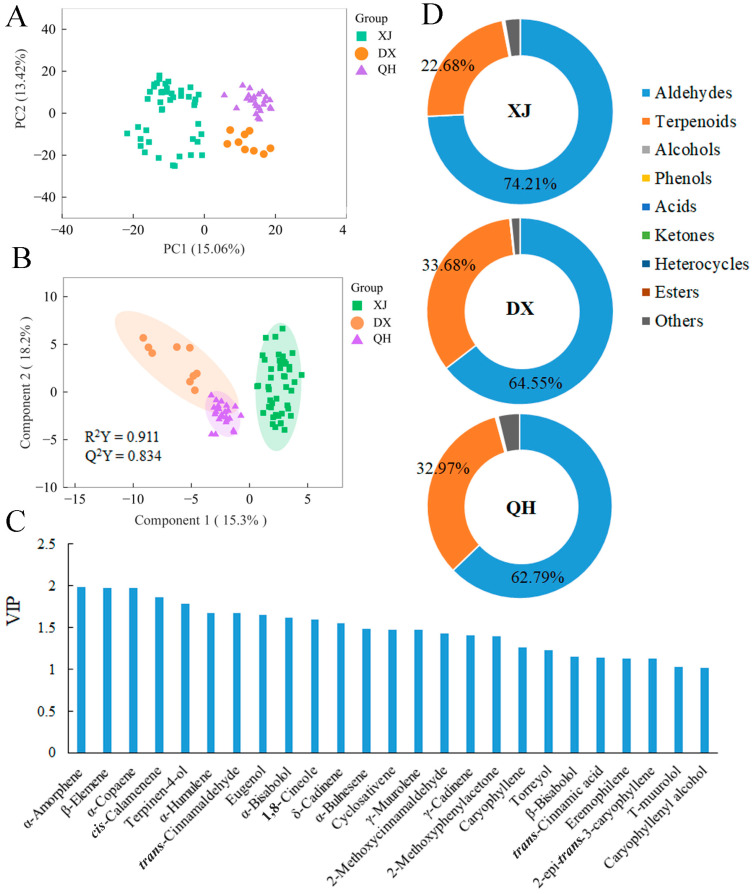
Multivariate analysis and chemical class distribution of volatile oils in *C. cassia* cultivars. (**A**) PCA score plot. (**B**) OPLS-DA score plot. (**C**) VIP plot derived from the OPLS-DA model. (**D**) Relative abundance of each chemical class in volatile oils. Note: Only the percentages for the top two categories are displayed. Abbreviations: XJ, Xijiang; DX, Dongxing; QH, Qinghua.

HS-SPME-GC-MS analysis of volatile oils from three *C. cassia* cultivars identified 71 differentially accumulated volatiles (DAVs) (Table 1). Total ion chromatograms (TICs) are shown in Figure S3, and cultivar-specific chromatograms are presented in Figure S4. PCA revealed distinct intra-cultivar clustering and inter-cultivar separation (Figure 4A), indicating significant differences in volatile profiles among the three cultivars. To further clarify inter-group variations, OPLS-DA was performed to extract variables responsible for the observed divergence. The OPLS-DA model exhibited excellent goodness (R^2^Y = 0.834) and predictive ability (Q^2^ = 0.911), confirming its robustness (Figure 4B). Based on the VIP scores, 25 DAVs with VIP > 1 were screened as potential discriminatory markers. To highlight the major discriminatory compounds, the top five compounds with the highest VIP values (ranked in descending order) were selected as key contributors, namely α-amorphene, β-elemene, α-copaene, *cis*-calamenene, and terpinen-4-ol (Figure 4C).

The distribution of DAVs across *C. cassia* cultivars is illustrated in Figure 4D. Aldehydes were the most abundant class of volatile compounds, accounting for 74.21%, 64.55%, and 62.79% of the total volatiles in XJ, DX, and QH, respectively. *Trans*-Cinnamaldehyde was the predominant aldehyde, representing 73.55%, 60.45%, and 57.56% of the total volatiles in the corresponding cultivar (Table S2). Terpenes were the second most abundant class, with proportions of 22.68% (XJ), 33.68% (DX), and 32.97% (QH). Notably, α-copaene content differed significantly among the three cultivars, being 3.88-fold and 2.02-fold higher in DX (17.49%) and QH (8.64%), respectively, compared to XJ (4.51%). Furthermore, δ-cadinene content was significantly higher in QH (6.07%) than in XJ (3.31%) and DX (4.64%) (Table S2).

### 3.5. Clustering Heatmap Analysis (HCA) of Volatile Components in C. cassia Cultivars

**Figure 5:**
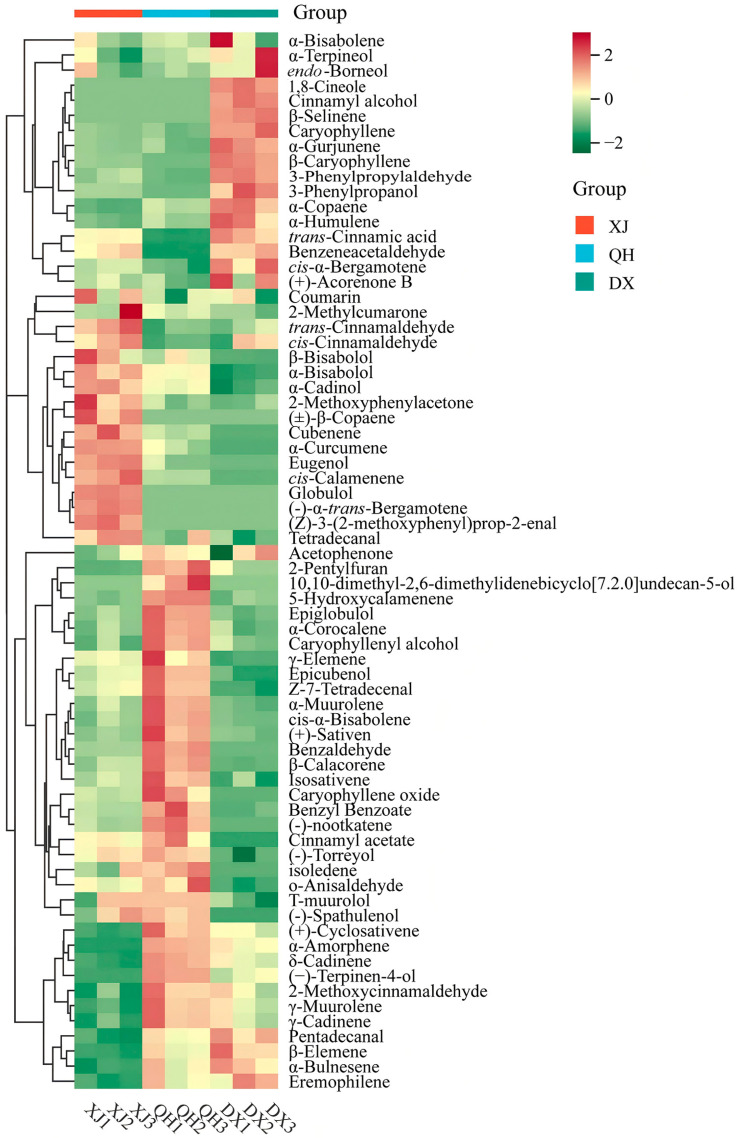
Hierarchical clustering analysis (HCA) heatmap of volatile compounds in *C. cassia* volatile oil. Columns represent cultivar accessions of XJ, DX, and QH; rows represent clustered volatile compounds. Red and green indicate high and low relative abundance levels, respectively. Abbreviations: XJ, Xijiang; DX, Dongxing; QH, Qinghua.

Based on the relative content (Table S2) of volatile compounds from XJ, DX, and QH samples, HCA grouped the 71 DAVs into four distinct clusters (Figure 5), revealing remarkable compositional differences in volatile profiles among the three cultivars. Cluster 1, predominantly enriched in DX, was dominated by sesquiterpenes and alcohols, with α-copaene, caryophyllene, and α-terpineol identified as characteristic compounds. Cluster 2 was predominantly accumulated in XJ samples, consisting of aldehydes, monoterpenes, and coumarins, including *trans*-cinnamaldehyde, *cis*-cinnamaldehyde, α-bisabolene, and coumarin. Cluster 3, composed of terpenes and esters, exhibited a dominant accumulation in QH, as exemplified by (+)-sativen, α-muurolene, and benzyl benzoate. Cluster 4 was commonly accumulated in both QH and DX, comprising terpenes and aldehydes, with γ-muurolene, γ-cadinene, and 2-methoxycinnamaldehyde as notable representatives.

### 3.6. Screening and Comparative Analysis of Aroma-Active Metabolites in C. cassia Cultivars

**Figure 6:**
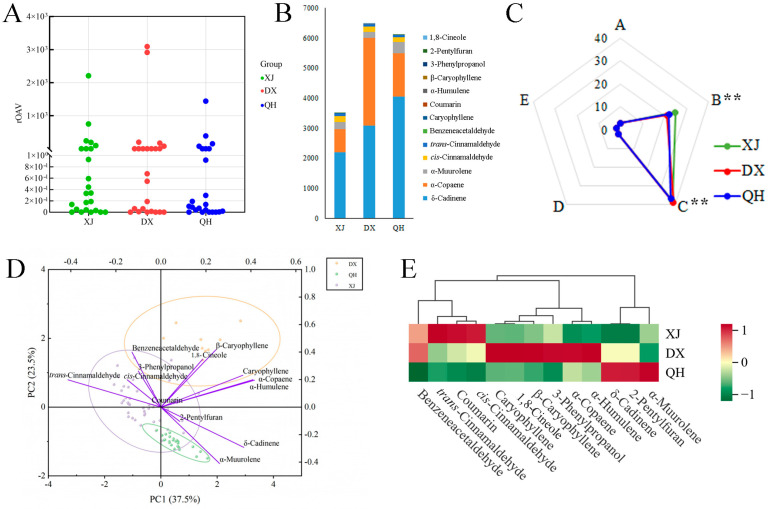
Analysis of aroma-active compounds in volatile oils from different *C. cassia* cultivars. (**A**) Scatter plot of rOAVs. (**B**) Thirteen shared aroma-active compounds (rOAV ≥ 1) among the three cultivars. (**C**) Classification of the 24 aroma-active compounds into five odor classes (A–E) based on their perceived aroma attributes. Significant differences among the three cultivar groups are indicated (**, *p* < 0.01). Class A: fresh and green scents; Class B: floral, fruity, and sweet scents; Class C: herbal and wood scents; Class D: a bake scent; and Class E: an unpleasant scent. (**D**) PCA biplot based on the 13 aroma-active compounds (rOAV ≥ 1). (**E**) HCA heatmap of the 13 key aroma-active compounds (OAV ≥ 1). In the heatmap, red and green denote high and low relative abundance levels, respectively. Abbreviations: XJ, Xijiang; DX, Dongxing; QH, Qinghua.

The rOAVs for 24 key aroma compounds were calculated by integrating their determined relative concentrations with reported odor threshold values from the literature (Figure 6A, Table 2). A total of 13 aroma-active compounds with rOAV ≥ 1 were shared across all three cultivars (Figure 6B). Notably, δ-cadinene, α-copaene, and α-muurolene exhibited the highest rOAVs and were thus identified as priority odorants for subsequent investigation. As shown in Figure 6C, the 71 previously identified DAVs were categorized into five classes (A–E) based on their odor characteristics. Among them, 3 components were assigned as Class A (fresh and green odors); 25 to Class B (floral, fruity, and sweet odors); 39 to Class C (herbal and woody odors); 2 to Class D (baked odor); and 2 to Class E (unpleasant odor).

ANOVA revealed that the number of class B (floral, fruity, and sweet odors) and class C (herbal and woody odors) compounds was significantly higher than that of classes A, D, and E (*p* < 0.05), highlighting their dominant contribution to the overall aromatic profile of *C. cassia*. Among the aroma-active compounds across cultivars, the four highest rOAV values were recorded for δ-cadinene (4044.35), α-copaene (2914.41), α-muurolene (390.30), and *trans*-cinnamaldehyde (98.07), all of which belonged to class C, confirming that class C volatiles are the principal drivers of the characteristic odor of *C. cassia*.

To identify cultivar-specific aroma signatures, pairwise comparisons were performed between each combination of the three cultivars. A total of 31, 36, and 35 differentially accumulated aroma-active metabolites were detected in XJ vs. DX (Table S3), XJ vs. QH (Table S4), and DX vs. QH (Table S5) comparisons, respectively. Venn analysis of these three pairwise groups identified 12 metabolites uniquely accumulated in XJ, including δ-cadinene, eugenol, and (±)-β-copaene. Notably, these 12 metabolites showed no significant differences between DX and QH, suggesting their potential as aroma-specific biomarkers for XJ. Similarly, 14 and 9 potential cultivar-specific aroma biomarkers were identified for DX and QH, respectively. Furthermore, eight common metabolites, including α-copaene, benzyl benzoate, and β-caryophyllene, were detected across all three cultivars (Table S6 and Figure S3).

### 3.7. Identification of Key Aroma-Active Compounds in C. cassia Cultivars Using PCA

**Figure 7:**
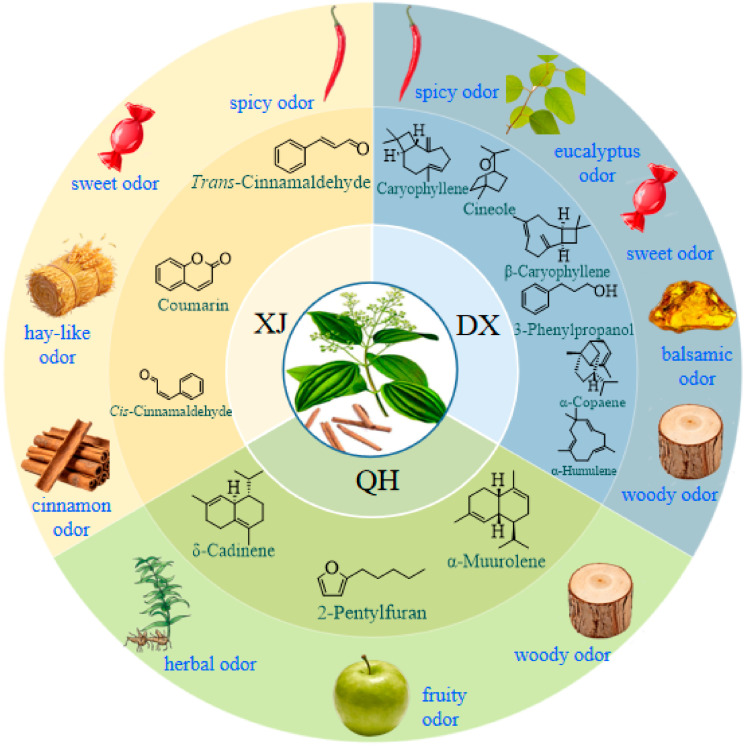
Key aroma-active compounds enriched in *C. cassia* cultivars. Abbreviations: XJ, Xijiang; DX, Dongxing; QH, Qinghua.

Aroma is a key determinant of essential oil quality in *C. cassia*. A 13 × 78 matrix was constructed using the 13 aroma-active compounds (rOAV ≥ 1) across 78 samples representing the three *C. cassia* cultivars, and PCA was performed with IBM SPSS Statistics 27. Four principal components (PCs) with eigenvalues > 1 were extracted, collectively explaining 79.03% of the total variance; the first two components accounted for 60.98% of the total variance (Table S7). The variables with the highest loadings on the first two PCs were α-humulene, *trans*-cinnamaldehyde, α-copaene, δ-cadinene, caryophyllene, β-caryophyllene, and α-muurolene (all with absolute loading values > 0.7). The PCA biplot (Figure 6D) illustrated distinct associations between aroma-active compounds and cultivar samples. In the upper-right quadrant of the plot, α-humulene, α-copaene, caryophyllene, and β-caryophyllene were clustered with DX samples. In the left section, *trans*-cinnamaldehyde and other related compounds were grouped with XJ samples. In the lower section, δ-cadinene, α-muurolene, and other aroma-active compounds showed a positive association with QH samples. HCA of the 13 aroma-active compounds (Figure 6E) further confirmed cultivar-specific odor signatures: *trans*-cinnamaldehyde (spicy), coumarin (sweet, hay-like), and *cis*-cinnamaldehyde (cinnamon-like) were most abundant in XJ; caryophyllene (spicy), 1,8-Cineole (eucalyptus-like), β-caryophyllene (sweet, woody, clove-like), 3-phenylpropanol (balsamic), and α-copaene and α-humulene (both woody) were enriched in DX, whereas 2-pentylfuran (fruity) and α-muurolene and δ-cadinene (both herbal) dominated in QH (Figure 7). Benzeneacetaldehyde was highly abundant in both XJ and DX cultivars.

## 4. Discussion

The integration of phenotypic evaluation, HS-SPME-GC-MS, and rOAV-PCA provided comprehensive insights into the aroma quality of three *C. cassia* cultivars (XJ, DX, and QH), effectively bridging morphological traits, volatile metabolomics, and sensory characteristics. By extracting and quantifying 71 VOCs across 78 samples from three different commercially predominant cultivars, we identified cultivar-specific markers such as *trans*-cinnamaldehyde (predominant in XJ, contributing to spicy notes via phenylpropanoid pathways) and α-muurolene (hallmark of QH, contributing to herbal notes linked to terpenoid biosynthesis pathways). These cultivar-specific markers not only validate the authenticity and flavor quality of *C. cassia* but also highlight biochemical crosstalk between phenotypic traits and volatile metabolism, where enzymatic transformations (e.g., the biosynthesis of *trans*-cinnamaldehyde in XJ and sesquiterpenes in DX/QH leading to distinct aroma profiles) amplify cultivar-specific sensory signatures. Differential accumulation of phenylpropanoids/terpenoids underpinned aroma divergence. PCA showed clear inter-cultivar clustering with minimal intra-cultivar variability, ensured by standardized analytical protocols for reliable data supporting flavor-oriented breeding.

Morphological and physiological traits are fundamental indicators for distinguishing plant cultivars, and combining phenotypic and chemical trait assessments is an efficient characterization approach [1]. Liang et al. [40] reported significant leaf phenotypic differences among *C. cassia* cultivars, and our preliminary research [41] further linked these differences to volatile oil content. Interestingly, no significant variations in three morphological traits (bark thickness, leaf length-width ratio, and leaf thickness) among XJ, DX, and QH cultivars were confirmed in this study, which could be attributed to environmental plasticity or a lack of correlation with secondary metabolite biosynthesis pathways. Soluble sugar levels were higher in QH and DX than in XJ, providing carbon skeletons for terpene and phenylpropanoid synthesis (e.g., δ-cadinene and cinnamaldehyde) [11], and soluble protein content followed the same pattern—this differed from the findings of Gao et al. [11], likely due to cultivation or methodological differences, emphasizing the need for standardized protocols. These metabolic divergences are mechanistically underpinned by cultivar-specific pathway regulation: XJ upregulates phenylalanine ammonia-lyase (PAL) and cinnamate-4-hydroxylase (C4H) for *trans*-cinnamaldehyde synthesis, while DX/QH enhances terpene synthase (TPS) and farnesyl diphosphate synthase (FPS) activity for sesquiterpenoid production [15]. Specialized metabolites closely related to volatile oil, cinnamaldehyde, and δ-cadinene also showed clear cultivar differences: QH had the highest volatile oil content, which was in line with previous studies [11,42], whereas XJ showed the highest *trans*-cinnamaldehyde content, and DX had the highest δ-cadinene content. These results further validate the cultivar differentiation and provide a basis for targeted breeding. This resolves a key limitation of earlier work, which has centered solely on volatile oil yield rather than connecting it to morphological and primary metabolic traits.

HS-SPME-GC-MS is a reliable technique for medicinal plant flavor analysis [43,44]. In *C. cassia*, several studies have demonstrated the utility of nontargeted metabolomes for analyzing metabolic profiles across different cultivars [11], growth years [14], and tissue types [45]. Given the remarkable advantages of the HS-SPME-GC-MS technique for volatile compound analysis, our previous study employed this approach to characterize the aroma profiles of bark samples harvested at different developmental stages [15]. In the present study, 71 DAVs were identified via HS-SPME-GC-MS analysis, with terpenes constituting the largest group, consistent with our previous findings [15]. This DAV number is substantially higher than the 42 reported by Gao et al. [11], likely due to our extended extraction time that enhances trace terpene capture for more precise intraspecific discrimination. Additionally, in line with the study by [46], aldehydes (mainly *trans*-cinnamaldehyde) accounted for the highest relative content of VOCs among the three cultivars (Figure 4), confirming their role as a conserved, taxonomically diagnostic marker. While prior work only validated this marker for interspecific differentiation, our data extend its utility by revealing cultivar-specific abundance variations that enable intraspecific identification. Hierarchical clustering revealed distinct cultivar VOC profiles: DX was enriched in 17 terpenoid DAVs, XJ in 17 unique aroma-active DAVs, and QH had the most exclusive DAVs, indicating metabolic specialization (Figure 5), consistent with the reported intraspecific metabolic polymorphism in aromatic plants such as citrus blend black tea [21] and *Citrus reticulata* ‘Chachi’ [17]. This divergence is linked to cultivar-specific transcriptional regulation: DX/QH’s terpenoid enrichment might correlate with TPS upregulation, while XJ’s aldehyde accumulation might align with PAL/C4H activity, establishing a clear gene-pathway–VOC link missing from prior studies. Such genotype-driven VOC divergence is consistent with molecular research on *C. cassia* cultivar differentiation [11,14,15]. Notably, unlike earlier studies that only described transcriptional differences, our work establishes a direct link between pathway regulation and VOC profiles, providing a mechanistic explanation for metabolic divergence rather than just phenotypic observation.

Aroma-active compounds in *C. cassia* vary by origin [22,47,48], but cultivar impacts on flavor remain underexplored. Aroma is determined by both concentration and odor threshold [27], and 24 aroma-active compounds were identified via rOAV values (Figure 6), representing core odorants for cultivar flavor differentiation. *Trans*-cinnamaldehyde (spicy odor) was the most abundant VOC but had moderate aromatic intensity due to its high odor threshold (0.75 mg/L) [28], while δ-cadinene (<5% relative content) had a significantly higher rOAV due to its ultra-low thresholds (0.0015 mg/L). This challenges the assumption that abundant compounds dominate aroma, and our study extends prior findings [1,14,45] by demonstrating that this rOAV-based dominance of trace terpenes is conserved across all three cultivars. Screening via rOAV (≥1) identified 13 major common aroma-active constituents with distinct cultivar distributions, and integrating rOAV rankings with PCA loadings confirmed seven key odorants for *C. cassia* essential oil, consistent with previous reports [22,28]. Unlike Gao et al. [11], who only quantified these key odorants, we link their rOAV variations to cultivar-specific pathway activity, explaining flavor divergence mechanistically. Cultivar-specific aroma profiles were delineated: XJ’s spicy profile stems from phenylpropanoid pathway prioritization, QH’s herbal–woody notes from enhanced sesquiterpene synthesis, and DX’s complex spicy–woody–sweet profile from coordinated activation of both pathways. Future research integrating gas chromatography–Olfactometry (GC-O) for sensory validation and transcriptomics for genetic mechanism elucidation would be highly valuable.

This methodology not only addresses gaps in authenticating *C. cassia* cultivars—where regional germplasm diversity and environmental confounding factors challenge traditional morphological identification—but also paves the way for hybrid systems integrating HS-SPME-GC-MS with emerging sensors (e.g., e-noses calibrated via our cultivar-specific VOC markers) for rapid, cost-effective on-site screening. By emphasizing volatile chemotaxonomy and pathway-driven metabolic profiles, it fortifies supply chain integrity against mislabeling of premium *C. cassia* varieties, fostering sustainable cultivation practices and consumer trust in high-quality medicinal and flavor markets.

### Limitations

A key limitation of this study lies in the inherent constraints of the HS-SPME-GC-MS-dominated analytical workflow: the technique has an extraction bias toward non-polar/moderately polar terpenes, which underrepresents polar VOCs; compound identification relies on commercial mass spectral libraries (NIST 2023), which may lead to misannotation of novel or cultivar-specific VOCs; and it also excludes non-volatile/semi-volatile metabolites, preventing the capture of critical precursors linked to aroma formation. To mitigate this single-technique limitation within the present study framework, we integrated the rOAV-PCA combined approach for flavor component evaluation, which enabled the systematic screening of core aroma-active compounds and the robust discrimination of cultivar-specific flavor profiles by complementing instrumental quantification with sensory relevance analysis and multivariate statistical validation. Moreover, the current workflow did not incorporate gas chromatography–ion mobility spectrometry (GC-IMS) and GC-O techniques, which would have added value to the present findings—GC-IMS could enhance the separation and detection of trace volatile isomers indistinguishable by GC-MS alone, while GC-O would enable direct correlation between instrumental data and human sensory perception, validating aroma-active roles beyond theoretical rOAV calculations. Additionally, the lack of transcriptomic and proteomic data limits full elucidation of molecular regulatory mechanisms, as genotype–metabolic phenotype links could only be inferred from metabolite abundances rather than direct gene expression or enzyme activity measurements. To address these remaining limitations, future research needs to integrate quantitative enzyme activity assays for core biosynthetic enzymes (e.g., PAL, TPS, and FPS) to establish more direct links between enzyme function and metabolic output without full multi-omics sequencing; we also plan to adopt GC-IMS and GC-O in follow-up analyses to further refine the volatile metabolome and sensory relevance of our results.

## 5. Conclusions

In conclusion, the aroma profiles of three major *C. cassia* cultivars were comprehensively characterized using HS-SPME-GC-MS. Key odorants were prioritized through an integrated, systematic approach combining rOAV calculation and multivariate statistical analysis. This approach definitively identified *trans*-cinnamaldehyde as the key spice-inducing compound in the XJ cultivar, a suite of sesquiterpenes (α-humulene, α-copaene, caryophyllene, and β-caryophyllene) as the primary contributors to the complex spicy–woody–sweet profile of DX, and δ-cadinene alongside α-muurolene as character-impact odorants that underpin the distinct herbal–woody note of QH. These findings not only provide a chemical basis for the sensory descriptors traditionally used to distinguish these commercial types but also offer a robust, chemistry-based toolkit for rapid cultivar authentication. Furthermore, the constructed phenotype–chemistry–sensory map offers an actionable scientific foundation for precision quality control, supply chain traceability, and aroma-directed breeding, ultimately enabling the development of targeted *C. cassia* varieties with tailored aroma profiles to meet specific market demands.

## Figures and Tables

**Table 1:** Profile of honey volatile compounds identified using GC-MS analysis.

RT ^1^	Volatile Compounds	Chemical Class	Kovat Index (KI) ^2^	
			Exp.	Lit.
8.483	Benzaldehyde	Aldehydes	962	960
9.005	2-Pentylfuran	Furans	991	991
9.706	1,8-Cineole	Alcohols	1030	1031
10.048	Benzeneacetaldehyde	Aldehydes	1049	1051
10.336	Acetophenone	Ketones	1065	1062
11.325	2-Methylcumarone	Ketones	1120	1123
12.045	3-Phenylpropylaldehyde	Aldehydes	1160	1160
12.225	*endo*-Borneol	Monoterpenes	1170	1171
12.423	(−)-Terpinen-4-ol	Monoterpenes	1181	1180
12.585	α-Terpineol	Monoterpenes	1190	1190
12.999	o-Anisaldehyde	Aldehydes	1213	1213
13.107	*cis*-Cinnamaldehyde	Aldehydes	1219	1219
13.358	3-Phenylpropanol	Alcohols	1233	1231
14.024	*trans*-Cinnamaldehyde	Aldehydes	1270	1270
14.78	Cinnamyl alcohol	Alcohols	1312	1312
15.679	Eugenol	Alcohols	1362	1357
15.823	(+)-Cyclosativene	Sesquiterpenes	1370	1370
15.931	α-Copaene	Sesquiterpenes	1376	1376
15.949	Isoledene	Alcohols	1377	1376
16.094	2-Methoxyphenylacetone	Ketones	1385	1385
16.291	(+)-Sativen	Sesquiterpenes	1396	1390
16.327	β-Elemene	Sesquiterpenes	1398	1391
16.525	α-Gurjunene	Sesquiterpenes	1409	1407
16.669	Isosativene	Sesquiterpenes	1417	1417
16.705	Caryophyllene	Sesquiterpenes	1419	1419
16.794	(±)-β-Copaene	Sesquiterpenes	1424	1430
16.956	γ-Elemene	Sesquiterpenes	1433	1433
16.992	(−)-α-*trans*-Bergamotene	Sesquiterpenes	1435	1437
17.082	Coumarin	Ethers	1440	1440
17.19	Cinnamyl acetate	Esters	1446	1440
17.208	(*Z*)-3-(2-methoxyphenyl)prop-2-enal	Aldehydes	1447	1447
17.28	*trans*-Cinnamic acid	Acids	1451	1450
17.334	α-Humulene	Sesquiterpenes	1454	1451
17.658	β-Caryophyllene	Sesquiterpenes	1472	1472
17.712	α-Curcumene	Sesquiterpenes	1475	1480
17.748	γ-Muurolene	Alcohols	1477	1477
17.802	α-Amorphene	Sesquiterpenes	1480	1483
17.91	β-Selinene	Sesquiterpenes	1486	1484
17.964	Eremophilene	Sesquiterpenes	1489	1488
17.982	*cis*-α-Bergamotene	Sesquiterpenes	1490	1490
18.144	α-Muurolene	Alcohols	1499	1499
18.234	*cis*-α-Bisabolene	Alcohols	1504	1504
18.252	2′-Methoxycinnamaldehyde	Aldehydes	1505	1505
18.252	α-Bulnesene	Sesquiterpenes	1505	1504
18.324	α-Bisabolene	Alcohols	1509	1505
18.36	(−)-nootkatene	Sesquiterpenes	1511	1511
18.396	γ-Cadinene	Sesquiterpenes	1513	1513
18.593	δ-Cadinene	Sesquiterpenes	1524	1525
18.683	*cis*-Calamenene	Sesquiterpenes	1529	1527
18.737	Cadina-1,4-diene	Sesquiterpenes	1532	1531
18.737	β-Calacorene	Sesquiterpenes	1532	1528
18.917	α-Calacorene	Sesquiterpenes	1542	1542
19.493	Caryophyllenyl alcohol	Alcohols	1574	1570
19.547	(−)-Spathulenol	Sesquiterpenes	1577	1577
19.619	Caryophyllene oxide	Sesquiterpenes	1581	1581
19.763	Epiglobulol	Sesquiterpenes	1589	1588
19.799	Globulol	Sesquiterpenes	1591	1592
19.907	*Z*-7-Tetradecenal	Aldehydes	1597	1597
20.159	Tetradecanal	Aldehydes	1611	1617
20.374	α-Corocalene	Sesquiterpenes	1623	1623
20.716	Epicubenol	Alcohols	1642	1647
20.716	T-muurolol	Alcohols	1642	1640
20.752	10,10-Dimethyl-2,6-dimethylenebicyclo[7.2.0]undecan-5β-ol	Alcohols	1644	1644
20.77	(−)-Torreyol	Alcohols	1645	1644
20.914	α-Cadinol	Sesquiterpenes	1653	1654
21.238	β-Bisabolol	Sesquiterpenes	1671	1671
21.472	α-Bisabolol	Sesquiterpenes	1684	1684
21.688	(+)-Acorenone B	Ketones	1696	1696
22.012	Pentadecanal	Aldehydes	1714	1712
22.857	Benzyl benzoate	Esters	1761	1760
23.577	5-Hydroxycalamenene	Sesquiterpenes	1801	1801

^1^ Retention Time (min). ^2^ KI: (Exp.) = experimental Kovats index; (Lit.) = literature Kovats index (using NIST libraries). Italic font was used for emphasis.

**Table 2:** The rOAV of differential volatile chemical components.

Sample	Odor Threshold (mg/L)	XJ	DX	QH	Threshold Reference
2-Pentylfuran	0.0060	0.1358	0.6790	1.2731	[31]
1,8-Cineole	0.0040	0.0000	1.6667	0.0000	[32]
Benzeneacetaldehyde	0.0040	2.4815	2.8704	0.1042	[31]
Benzaldehyde	0.3500	0.0019	0.0000	0.0052	[22]
Acetophenone	0.0650	0.1880	0.1880	0.1902	[30]
*endo*-Borneol	0.1800	0.3379	0.5453	0.2955	[28]
(−)-Terpinen-4-ol	0.5900	0.0008	0.0138	0.0179	[33]
α-Terpineol	0.3000	0.0402	0.0623	0.0394	[29]
*cis*-Cinnamaldehyde	0.0050	194.0074	172.2593	153.4444	[34]
3-Phenylpropanol	0.0030	0.5926	1.7284	0.0000	[35]
o-Anisaldehyde	0.6300	0.0303	0.0088	0.0392	[36]
*trans*-Cinnamaldehyde	0.7500	98.0663	80.6064	76.7504	[28]
Cinnamyl alcohol	1.0000	0.0002	0.0022	0.0000	[34]
Eugenol	0.0300	0.4432	0.0000	0.0880	[38]
α-Copaene	0.0060	751.8457	2914.4136	1440.7176	[22]
Caryophyllene	0.1600	0.9306	2.2269	0.9193	[28]
Coumarin	0.0250	1.7333	1.4000	1.2111	[22]
Cinnamyl acetate	0.1500	0.0519	0.0000	0.0694	[30]
α-Humulene	0.1200	1.4571	4.1296	2.0475	[36]
β-Caryophyllene	0.0270	0.3320	2.8532	0.0000	[38]
α-Muurolene	0.0110	240.2929	201.3805	390.3030	[38]
δ-Cadinene	0.0015	2209.0123	3091.9753	4044.3519	[29]
Caryophyllene oxide	5.0000	0.000044	0.000000	0.000000	[37]
α-Bisabolol	1.3000	0.1715	0.0610	0.1359	[39]

Italic font was used for emphasis.

## Data Availability

The original contributions presented in the study are included in the article, and further inquiries can be directed to the corresponding author/Supplementary Materials.

## Supplementary Materials

The following supporting information can be downloaded at: https://www.mdpi.com/article/10.3390/foods15040723/s1, Figure S1: GC-MS chromatograms of mixed reference standards (A) and essential oil sample (B). 14: *trans*-cinnamaldehyde, 44: δ-cadinene; Figure S2: Standard curve of *trans*-cinnamaldehyde (A) and δ-cadinene (B); Figure S3: Total ion chromatogram (TIC) of GC-MS analysis; Figure S4: GC-MS chromatogram of essential oil from 78 batches of *C. cassia*. (A) XJ. (B) DX. (C) QH; Figure S5: Venn analysis of differential metabolites across the three cultivar comparison groups; Table S1: The Sampling situation of *C. cassia*; Table S2: The odor classification of the 71 volatile compounds and their relative content (%) in three *C. cassia* cultivars; Table S3: The differential volatile chemical components between XJ and QH; Table S4: The differential volatile chemical components between DX and QH; Table S5: The differential volatile chemical components between XJ and DX; Table S6: The specific and common differential accumulated metabolites of different tissues detected by GC-MS in *C. cassia*; Table S7: The eigenvalues, load matrix, variance contribution rate, and cumulative variance contribution rate of principal components.

## Abbreviations

XJ	Xijiang
DX	Dongxing
QH	Qinghua
HS-SPME	Headspace solid-phase microextraction
GC-MS	Gas chromatography–mass spectrometry
rOAV	Relative odor activity value
PCA	Principal component analysis
CH.P	The Pharmacopeia of the People’s Republic of China
VOCs	Volatile organic compounds
SD	Standard deviation
ANOVA	One-way analysis of variance
HCA	Hierarchical cluster analysis
VIP	Variable importance in projection
OPLS-DA	Orthogonal partial least squares discriminant analysis
Log_2_FC	Log_2_ fold change
DAVs	Differentially accumulated volatiles
TICs	Total ion chromatograms
GC-O	Chromatography–olfactometry
