# Supplementary material for: Comparative Analysis of Volatile Compounds and Characterization of Key Flavor Compounds in Cinnamomum cassia Barks of Different Cultivars
Source: Foods. 2026 Feb 15;15(4):723. doi: 10.3390/foods15040723 (PMC12940784; doi:10.3390/foods15040723)
Supplement: Supplementary file 1 [file foods-15-00723-s001.zip › Supplementary Material.pdf]

Supplementary Material

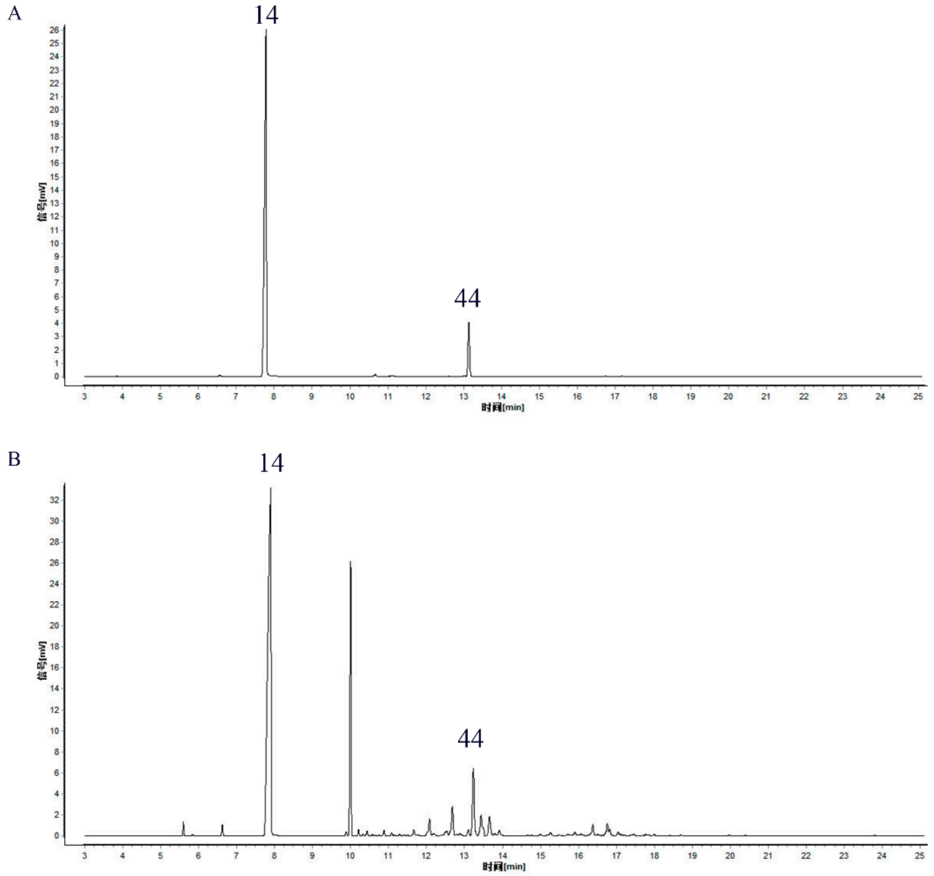

Figure S1. GC-MS chromatograms of mixed reference standards (A) and essential oil sample (B). 14: *trans*-cinnamaldehyde 44: δ-cadinene.

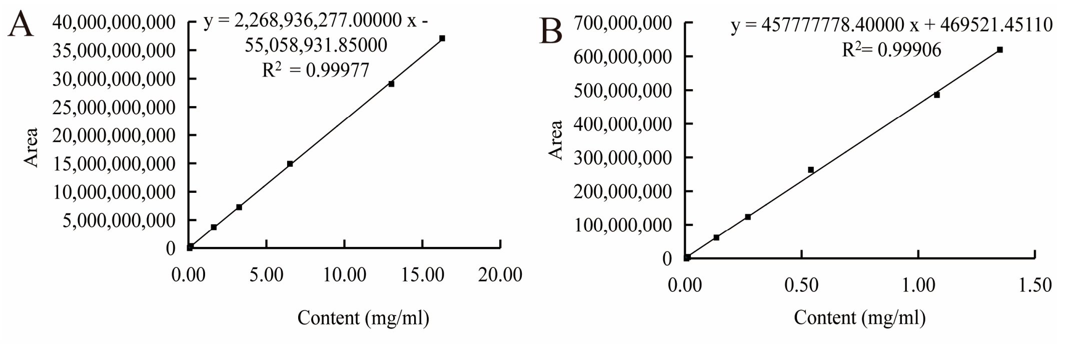

Figure S2. Standard curve of *trans*-cinnamaldehyde (A) and δ-cadinene (B).

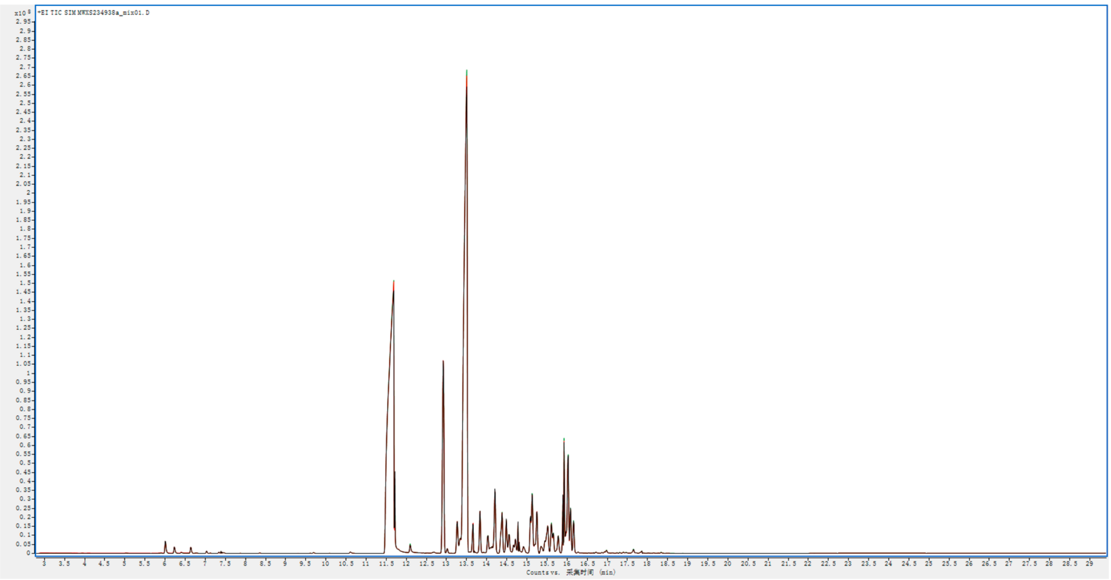

Figure S3. Total ion chromatogram (TIC) of GC-MS analysis.

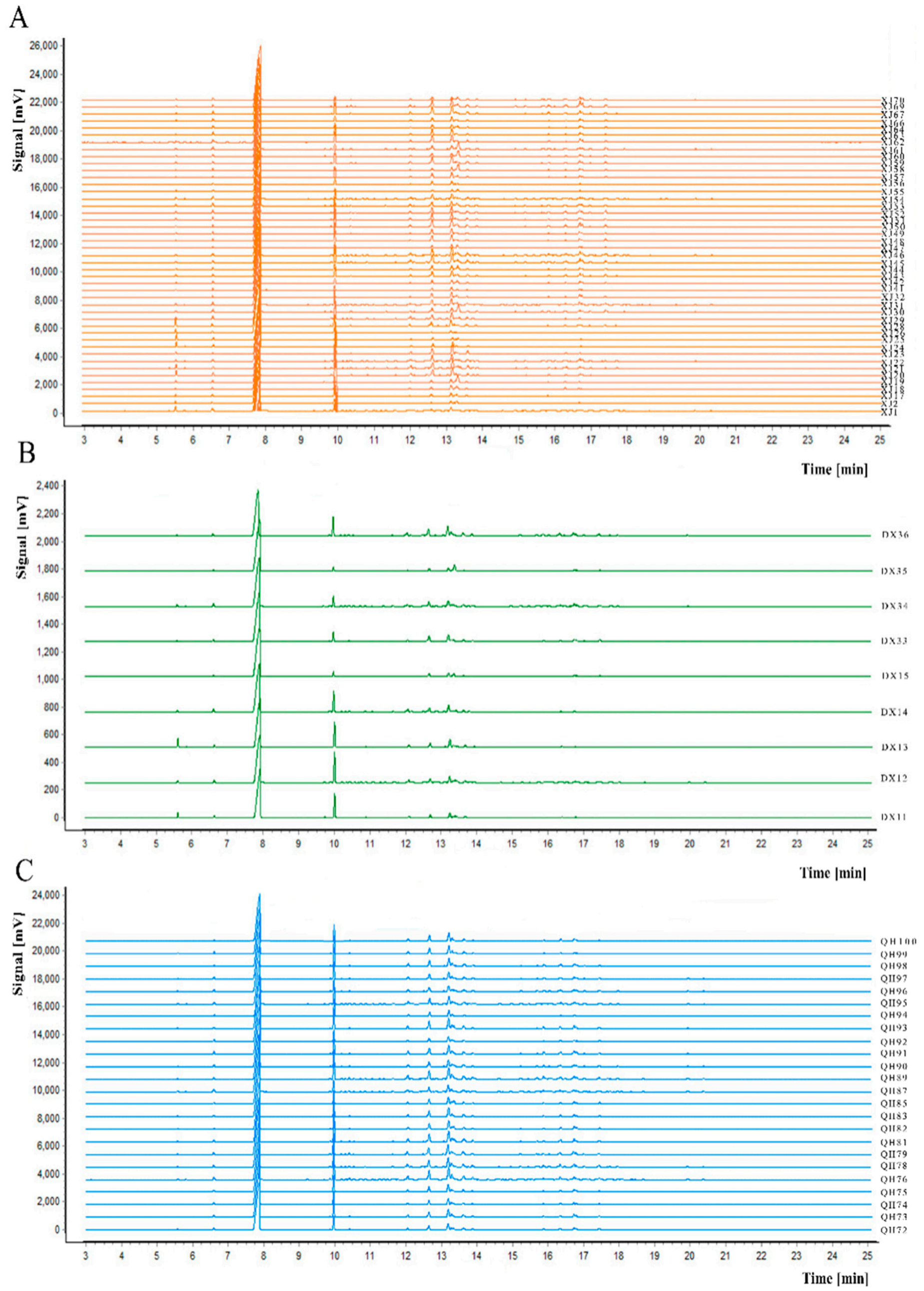

Figure S4. GC-MS chromatogram of essential oil from 78 batches of of *C. cassia*. (A) XJ. (B) DX. (C) QH.

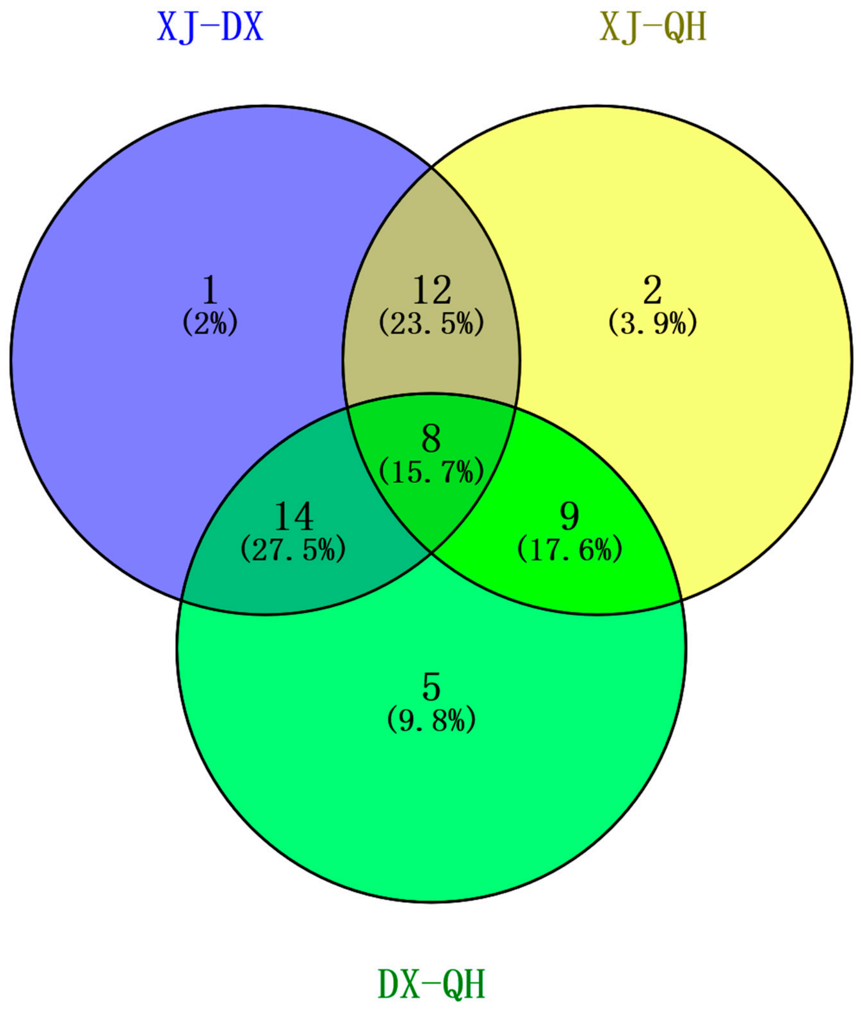

Figure S5. Venn analysis of differential metabolites across the three cultivar comparison groups.
